# Proteomics-Derived Biomarker Panel Facilitates Distinguishing Primary Lung Adenocarcinomas With Intestinal or Mucinous Differentiation From Lung Metastatic Colorectal Cancer

**DOI:** 10.1016/j.mcpro.2024.100766

**Published:** 2024-04-10

**Authors:** Jiaying Liu, Xiaona Chang, Liujia Qian, Shuo Chen, Zhangzhi Xue, Junhua Wu, Danju Luo, Bo Huang, Jun Fan, Tiannan Guo, Xiu Nie

**Affiliations:** 1 Department of Pathology, Union Hospital, Tongji Medical College, Huazhong University of Science and Technology, Wuhan, China; 2 Center for ProtTalks, Westlake Laboratory of Life Sciences and Biomedicine, Key Laboratory of Structural Biology of Zhejiang Province, School of Life Sciences, Westlake University, Hangzhou, Zhejiang, China; 3 Institute of Basic Medical Sciences, Westlake Institute for Advanced Study, Hangzhou, Zhejiang, China; 4 Research Center for Industries of the Future, Westlake University, Hangzhou, Zhejiang, China

**Keywords:** primary lung adenocarcinomas, intestinal or mucinous differentiation, lung metastatic colorectal cancer, proteomics, differential diagnosis

## Abstract

The diagnosis of primary lung adenocarcinomas with intestinal or mucinous differentiation (PAIM) remains challenging due to the overlapping histomorphological, immunohistochemical (IHC), and genetic characteristics with lung metastatic colorectal cancer (lmCRC). This study aimed to explore the protein biomarkers that could distinguish between PAIM and lmCRC. To uncover differences between the two diseases, we used tandem mass tagging–based shotgun proteomics to characterize proteomes of formalin-fixed, paraffin-embedded tumor samples of PAIM (n = 22) and lmCRC (n = 17).Then three machine learning algorithms, namely support vector machine (SVM), random forest, and the Least Absolute Shrinkage and Selection Operator, were utilized to select protein features with diagnostic significance. These candidate proteins were further validated in an independent cohort (PAIM, n = 11; lmCRC, n = 19) by IHC to confirm their diagnostic performance. In total, 105 proteins out of 7871 proteins were significantly dysregulated between PAIM and lmCRC samples and well-separated two groups by Uniform Manifold Approximation and Projection. The upregulated proteins in PAIM were involved in actin cytoskeleton organization, platelet degranulation, and regulation of leukocyte chemotaxis, while downregulated ones were involved in mitochondrial transmembrane transport, vasculature development, and stem cell proliferation. A set of ten candidate proteins (high-level expression in lmCRC: CDH17, ATP1B3, GLB1, OXNAD1, LYST, FABP1; high-level expression in PAIM: CK7 (an established marker), NARR, MLPH, S100A14) was ultimately selected to distinguish PAIM from lmCRC by machine learning algorithms. We further confirmed using IHC that the five protein biomarkers including CDH17, CK7, MLPH, FABP1 and NARR were effective biomarkers for distinguishing PAIM from lmCRC. Our study depicts PAIM-specific proteomic characteristics and demonstrates the potential utility of new protein biomarkers for the differential diagnosis of PAIM and lmCRC. These findings may contribute to improving the diagnostic accuracy and guide appropriate treatments for these patients.

## Highlights

• Proteomic features of primary lung adenocarcinomas with intestinal or mucinous differentiation (PAIM) and lung metastatic colorectal cancer (lmCRC) are explored.
• 105 proteins out of 7871 were significantly dysregulated between PAIM and lmCRC samples and well-separated two groups by UMAP.
• Five protein biomarkers including CDH17, CK7, MLPH, FABP1, and NARR were effective biomarkers for distinguishing PAIM from lmCRC.

## Graphical Abstract

**Figure:**
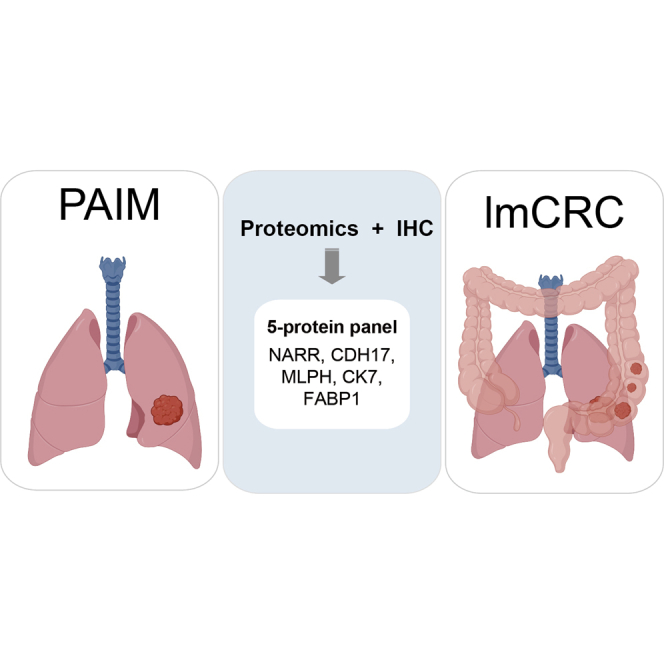

## In Brief

The diagnosis of primary lung adenocarcinomas with intestinal or mucinous differentiation (PAIM) remains challenging due to the overlapping histomorphological, immunohistochemical, and genetic characteristics with lung metastatic colorectal cancer (lmCRC). In this study, we used LC-MS/MS to characterize proteomes of PAIM and lmCRC. Then three machine learning algorithms were utilized to screen for differential proteins with diagnostic significance. The identified candidate proteins (CDH17, FABP1 highly expressed in lmCRC; NARR, MLPH, CK7 highly expressed in PAIM) were robustly validated in an independent cohort by IHC.

Accurate differentiation of primary tumors *versus* metastatic ones is critical for effective cancer diagnosis and appropriate treatment decisions, as it directly influences prognosis. Although lung adenocarcinomas are generally distinguishable from gastrointestinal cancers, certain cases exhibit overlapping histomorphological, immunohistochemical, and molecular characteristics. The 2021 World Health Organization classification of lung tumors recognizes three subtypes of primary lung adenocarcinomas with intestinal or mucinous differentiation (PAIM), namely enteric-type adenocarcinoma, invasive mucinous adenocarcinoma (IMA), and colloid adenocarcinoma (1). Due to their morphological and immunohistochemistry (IHC) similarities, it is essential to differentiate these subtypes from metastatic carcinomas originating from extrapulmonary sites.

Enteric-type adenocarcinoma is a primary pulmonary adenocarcinoma resembling colorectal adenocarcinoma, characterized by acinar, cribriform, or papillotubular structures with common intraluminal necrotic cellular debris, tall columnar tumor cells, as well as focal mucin production (1, 2, 3). IMA is another primary lung adenocarcinoma subtype, displaying goblet and/or columnar cell morphology with abundant intracytoplasmic mucin and small, basally oriented nuclei (1, 4). Owing to their analogous morphology, IMAs must be carefully distinguished from metastatic mucinous adenocarcinoma originating from other sites, including the gastrointestinal tract, pancreatobiliary system, and ovary. The high expression of CK20 and CDX2 can aid in this differentiation from mucinous colorectal adenocarcinomas, although their specificity is limited. Colloid adenocarcinoma shows abundant extracellular mucin in pools that expand alveolar spaces and disrupt their walls (1, 5). In this study, we collectively refer to these PAIM. In summary, PAIM and lung metastatic colorectal cancer (lmCRC) share similar histomorphological features, posing a challenge for accurate identification based solely on histomorphological traits.

In terms of immunoprofiling, there remains a scarcity of dependable IHC markers to differentiate between PAIM and lmCRC. Clinically, CK7 and TTF-1 are typical IHC markers for lung adenocarcinoma (2, 6). However, TTF-1 expression is frequently absent in PAIM (2, 7, 8). Furthermore, positive CK7 expression can also be detected in a minority of colorectal cancer (CRC) cases and some CRC metastases to the lung exhibit positive TTF-1 expression (9, 10, 11). These limitations have compounded the challenges in differential diagnosis. To enhance diagnostic accuracy, various other IHC markers have been investigated, including Villin, SATB2, β-catenin, and CDH17, but their differential diagnostic performance warrants further evaluation (12, 13).

At present, distinguishing between PAIM and lmCRC based solely on routine histomorphological and IHC profiles is challenging. The accurate differential diagnosis necessitates a comprehensive assessment that incorporates a detailed clinical history and extensive imaging examinations such as PET/computed tomography (CT) and gastrointestinal endoscopy. However, some patients may have concerns about the complexity, time consumption, cost, and potential stress of the comprehensive examination process, potentially resulting in missed optimal treatment opportunities and adverse outcomes. Nevertheless, it is crucial to highlight that this thorough assessment is now a standard of care at most leading cancer centers. Therefore, there is an urgent need for more reliable methods to differentiate PAIM from lmCRC.

Multiple studies have been conducted on the diagnostic potential of genetic features (14, 15, 16, 17, 18, 19, 20). However, studies focusing on genomic events, such as mutations, have failed to provide a reliable differential diagnostic strategy, as PAIM and lmCRC often exhibit similar genomic alterations, including frequent KRAS mutations (14, 15, 16, 17, 18). In addition, although diagnostic classifiers based on DNA methylation profiles can reliably distinguish enteric-type adenocarcinoma from lmCRC (19, 20), the differentially methylated regions identified by two research teams rarely overlap. This observation underscores the inherent limitations of epigenomic data, which is highly dependent on the platform used in the analysis. Moreover, the epigenetic and RNA expression patterns of IMA closely resemble those of upper gastrointestinal cancers (20, 21). Given the limitations in the clinical applicability of methylation-based classifiers, there is a pressing need to identify more practical and dependable biomarkers to differentiate PAIM from lmCRC.

Proteins play a pivotal role as the primary executors guiding cellular activities. Therefore, an in-depth characterization of the proteome is essential for a comprehensive understanding of disease processes (22, 23). To date, several studies have investigated lung adenocarcinoma from a proteomics perspective, contributing valuable insights to researchers and clinicians seeking to improve diagnosis and treatment of the disease (24, 25). Moreover, researchers have developed reliable diagnostic strategies based on novel markers identified through proteomics (26, 27, 28, 29, 30, 31). However, to the best of our knowledge, no studies have yet investigated the proteomic features of PAIM or examined the differentially expressed proteins (DEPs) between PAIM and lmCRC, utilizing proteomics approaches.

In this study, we collected formalin-fixed, paraffin-embedded (FFPE) tumor samples from 22 PAIM patients and 17 lmCRC patients according to existing diagnostic criteria. We then performed in-depth proteomics analysis using tandem mass tagging (TMT)-based shotgun proteomics, identifying 105 DEPs when comparing the two groups. Then three machine learning algorithms, namely support vector machine (SVM), random forest, and the Least Absolute Shrinkage and Selection Operator (LASSO), were utilized to select and rank protein features by their diagnostic significance. The five selected proteins were further validated in an independent cohort by IHC to confirm their ability to distinguish PAIM from lmCRC.

## Experimental Procedures

### Patient Cohorts and Diagnosis

This study was approved by the Ethics Committees of the Union Hospital, Tongji Medical College, Huazhong University of Science and Technology (ethical approval number S377), and written consent forms were obtained from all patients. All procedures conformed to the principles of the Declaration of Helsinki.

Our study included 22 patients with PAIM (one colloid adenocarcinoma, three IMAs, and 18 enteric-type adenocarcinomas) and 17 patients with lmCRC in the discovery cohort. All patients were adults, who underwent lung resection surgery at the Union Hospital, Tongji Medical College, Huazhong University of Science and Technology between September 2014 and January 2022, and had available FFPE tumor samples. We also included a validation cohort consisting of surgical and biopsy samples from 11 PAIM (one IMA and ten enteric-type adenocarcinomas) and 19 lmCRC patients, which is more in line with clinical practice needs. Each patient was reevaluated and classified as either PAIM or lmCRC according to the classification criteria of the 2021 World Health Organization guidelines and clinical consensus by two experienced pathologists. The diagnostic criteria for PAIM include intestinal or mucinous differentiation morphology, expression of IHC markers for intestinal differentiation (CDX2, CK20, and Villin), and exclusion of metastasis from colorectal carcinoma based on medical history, endoscopy, or PET/CT, as well as follow-up. The diagnosis of lmCRC was mainly based on the detection of synchronous or metachronous CRC. The researchers were blinded to the diagnosis grouping when assessing the validation cohort. The clinical data including sex, age at diagnosis, height, weight, BMI, systolic and diastolic pressure, smoking status, alcohol history, tumor location, tumor maximum dimension, tumor staging (American Joint Committee on Cancer eighth edition), and IHC results (TTF-1, Napsin A, CK7, CK20, Villin, and CDX2) were collected from the medical records. Patient details and clinicopathological characteristics are summarized in supplemental Table S1.

### Experimental Design and Statistical Rationale

**Fig. 1:**
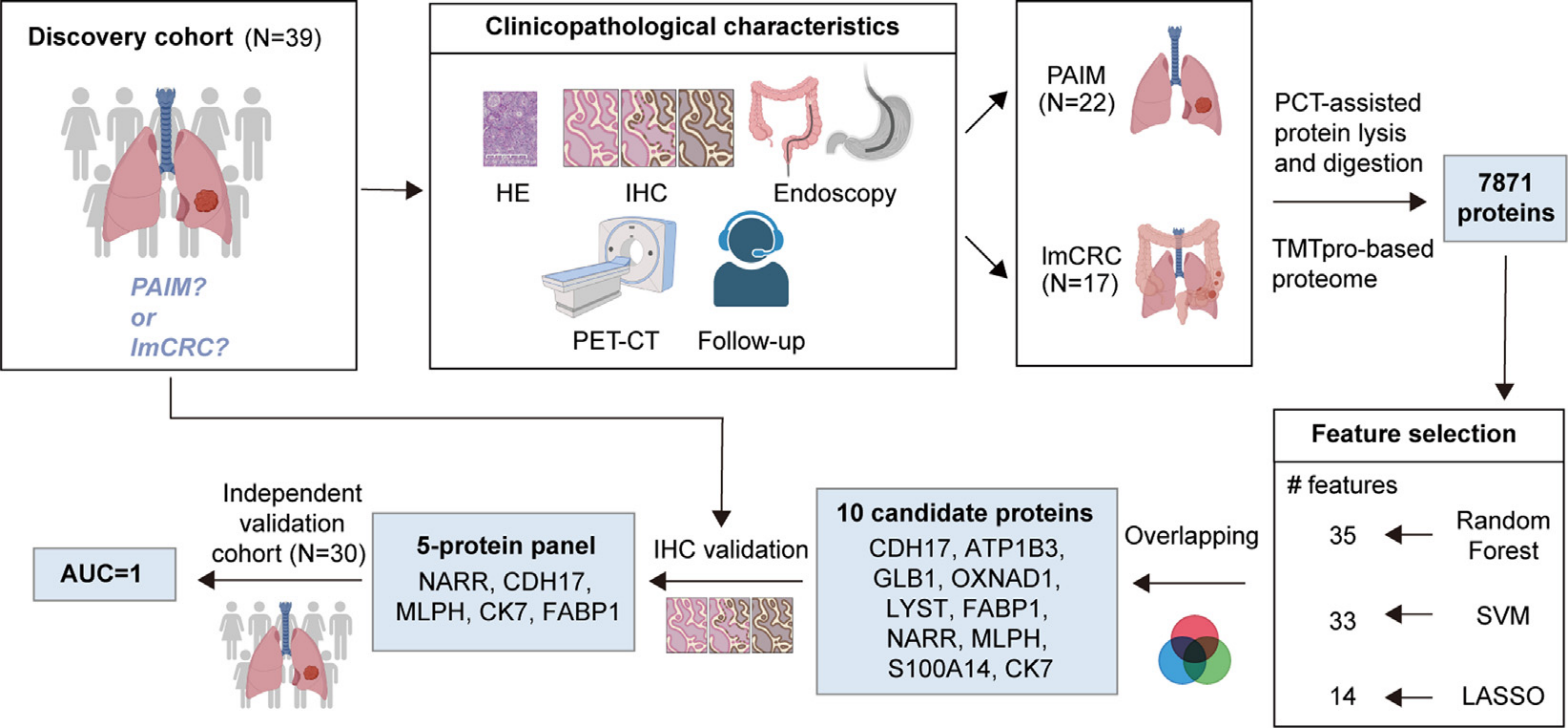
**Graphical summary of the study design and data analyses.** FFPE tumor samples from 22 patients with PAIM and 17 patients with lmCRC were collected as the discovery cohort. Clinicopathological characteristics were analyzed between two groups. We performed multidimensional LC-MS/MS to characterize proteomes of PAIM and lmCRC. Then machine learning algorithms (SVM, random forest, and LASSO) were utilized to pick out differential proteins with diagnostic significance. A set of five candidate proteins was ultimately determined to distinguish PAIM and lmCRC based on immunohistochemical (IHC) results in the discovery cohort. These candidate proteins were further validated in an independent cohort (11 PAIM and 19 lmCRC patients) by IHC to confirm their ability to distinguish PAIM from lmCRC. FFPE, formalin-fixed, paraffin-embedded; LASSO, Least Absolute Shrinkage And Selection Operator; lmCRC, lung metastatic colorectal cancer; PAIM, primary lung adenocarcinomas with intestinal or mucinous differentiation; SVM, support vector machine.

This study included four phases (Fig. 1): (1) FFPE-pressure cycling technology (PCT)-TMT–based proteomics, (2) bioinformatic analyses, (3) feature selection, and (4) IHC validation.

Phase 1 involved the collection of samples from PAIM and lmCRC patients. Pathologists evaluated and classified patients based on the classification criteria. The samples were punched (diameter 1 mm) from the area of interest of FFPE blocks, thus ensuring that cancerous tissues in the sample accounted for more than 85%. A total of 22 tissue cores from 22 PAIM patients and 17 tissue cores from 17 lmCRC patients were collected. Peptides were generated from each of the 39 specimens using PCT-based protein extraction and digestion. TMT-based proteomics workflow was employed to process 39 peptide samples, six randomly selected technical replicates (including three intrabatch and three interbatch replicates), and three common pooled samples. Data quality control was performed by analyzing the reproducibility of proteomic data between technical replicates and among the pooled samples to ensure the stability of TMT labeling and the mass spectrometer.

In phase 2, DEPs between PAIM (n = 22) and lmCRC (n = 17) groups were screened using an unpaired two-sided Welch's *t* test. Significance was determined using a Benjamini–Hochberg (B–H) adjusted *p* value threshold of less than 0.05 and a |log2FC| larger than 0.25. Pathway enrichment analysis for the resulting 105 DEPs was conducted using Metascape.

Phase 3 aimed at identifying significant proteins that can differentiate between PAIM and lmCRC. Three algorithms, namely SVM, random forest, and LASSO (32, 33, 34), were employed for feature selection. Missing values (5.7% of total) were imputed using 0.8 times the minimum value of the whole protein matrix. Firstly, a SVM classifier was built based on all quantified proteins with default parameters by R package geNetClassifier (28, 32). Secondly, 105 aforementioned DEPs were further selected by random forest analysis (33). The protein matrix was normalized using *Z*-score. By the R package randomForest, we chose 35 proteins with mean decrease accuracy values of over three in 39 samples as input features to build one thousand trees. We randomly divided the dataset into a training set and a test set at a ratio of 4:1 through the hold-out method. We performed 50 iterations of hold-out method to generate multiple classifiers using protein features with mean decrease accuracy greater than three in each training set. The final performance was evaluated by the accuracy and area under curve (AUC) in a receiver operating characteristic curve (ROC) of the test set. We kept each iteration that obtained both accuracy and AUC of 1 to select the most informative protein features for accurate diagnosis. Thirdly, a LASSO classifier was built based on all quantified proteins with default parameters by R package caret (34). We performed a 10-fold cross-validation to identify the minimum lambda value and its corresponding one standard error, which allowed us to select the most stringent set of biomarkers. After employing the SVM, random forest, and LASSO algorithms, we determined a set of ten candidate proteins (CDH17, ATP1B3, GLB1, OXNAD1, CK7, FABP1, NARR, MLPH, S100A14, and LYST), which were identified by two or more of the algorithms we employed, to effectively distinguish between PAIM and lmCRC.

In the last phase, we performed IHC analysis of the nine candidate proteins in our discovery cohort. From these nine proteins, we selected the five proteins (CDH17, CK7, MLPH, FABP1, and NARR) with good diagnostic performance for further evaluation in our validation cohort.

### Proteomics Analysis

Thirty-nine patients from the discovery cohort were randomly distributed into three batches (supplemental Table S2). Proteins were extracted from FFPE tissue specimens of these patients and digested by PCT–assisted proteomic sample preparation as described previously (35, 36). Peptide samples were labeled with TMTpro 16plex reagent (Thermo Fisher Scientific) (supplemental Table S2). The combined sample of each batch was fractionated as described previously (36). A total of 60 fractions were collected by high-pH reversed-phase fractionation and concatenated into 30 fractions. After dried under vacuum, peptides were separated in a 60-min gradient by Thermo Fisher ScientificTM UltiMateTM 3000 RSLCnano System and analyzed using an Orbitrap Exploris 480 mass spectrometry with FAIMS Pro interface in data-dependent acquisition mode. The detailed settings of mass spectrometry have been described previously (37). The raw data were analyzed by Proteome Discoverer software (version 2.4.0.305, Thermo Fisher Scientific, https://thermo.flexnetoperations.com/control/thmo/download?element=11324157) against a FASTA file containing 20,368 reviewed *Homo sapiens* proteins downloaded from UniProt website on 15 July 2020 as described previously (36). Briefly, trypsin was chosen as the enzyme for peptide generation, allowing a maximum of two missed cleavages. The mass tolerance for precursor ions was set at 10 ppm, while the tolerance for fragment ions was set at 0.02 Da. Variable modifications included oxidation (M) and acetyl (protein N terminus), while carbamidomethyl (C) was set as a static modification. Further analysis was conducted on proteins and peptides with false discovery rates below 1% that were quantified.

### Quality Control

Outliers of protein abundance ratio were defined according to Tukey’s fences, where k equals 2. The outliers (2.6% of total) were imputed as 1.657, which is the thus computed cut-off for determining outliers. The reproducibility of proteomic data was evaluated by CVs among common pool samples and technical replicates. The CVs among common pool samples were calculated by the abundance of quantified proteins in each pool sample, while the CVs between technical replicates were calculated by the abundance ratios.

### Differentially Expressed Proteins

Differential analysis of proteomic data between PAIM and lmCRC groups were performed by unpaired two-sided Welch's *t* test. The *p* values were corrected by the B–H procedure. FC of each protein was calculated by dividing the mean of abundance ratio in lmCRC group by those in PAIM group. The DEPs were selected when the B–H adjusted *p* value was less than 0.05 and |log_2_FC| was larger than 0.25.

### Protein Markers for Classification Screening

To identify significant proteins that can distinguish between PAIM and lmCRC, we employed three different algorithms, namely SVM, random forest, and the LASSO (32, 33, 34). The missing values were imputed using the 0.8∗minimum of whole protein matrix. Firstly, a SVM classifier was built based on all quantified proteins with default parameters by R package geNetClassifier (28, 32). Secondly, 105 aforementioned DEPs were further selected by random forest analysis (33). The protein matrix was normalized using *Z*-score. By the R package randomForest, we chose 35 proteins with mean decrease accuracy values of over three in 39 samples as input features to build 1000 trees. We randomly divided the dataset into a training set and a test set at a ratio of 4:1 through the hold-out method. We performed 50 iterations of hold-out method to generate multiple classifiers using protein features with mean decrease accuracy greater than three in each training set. The final performance was evaluated by the accuracy and AUC in a ROC of the test set. We kept each iteration that obtained both accuracy and AUC of 1 to select the most informative protein features for accurate diagnosis. Thirdly, a LASSO classifier was built based on all quantified proteins with default parameters by R package caret (34). We performed a 10-fold cross-validation to identify the minimum lambda value and its corresponding one standard error, which allowed us to select the most stringent set of biomarkers. After employing the SVM, random forest, and LASSO algorithms, we determined a set of ten candidate proteins (CDH17, ATP1B3, GLB1, OXNAD1, CK7, FABP1, NARR, MLPH, S100A14, and LYST), which were identified by two or more of the algorithms we employed, to effectively distinguish between PAIM and lmCRC.

### IHC Analysis

Since commercially available antibodies for LYST were not available, we performed IHC analysis of the nine candidate proteins in our discovery cohort. From these nine proteins, we selected the five proteins (CDH17, CK7, MLPH, FABP1, and NARR) with good diagnostic performance for further evaluation in our validation cohort. All patients had hematoxylin and eosin slides and IHC stains at the time of primary diagnosis. In this study, IHC staining for candidate proteins was performed according to standard clinical operating procedures. Briefly, 3-μm thick FFPE sections were subjected to routine IHC analysis. Appropriate positive and negative controls on each slide were used for quality control. Detailed assay and antibody information, as well as scoring method were provided in supplemental Table S3. The IHC results were judged by the histological score (Hscore, range: 0–300). The Hscore was calculated as follow: Hscore = (percentage of tumor cells of weak intensity × 1) + (percentage of tumor cells of moderate intensity × 2) + (percentage of tumor cells of strong intensity × 3) (38).

### Statistical Analysis

All statistical analyses and data visualization were performed using R (version 4.1.2, https://mirror.lzu.edu.cn/CRAN/src/base/R-4/R-4.1.2.tar.gz), including the geNetClassifier, randomForest, caret, Uniform Manifold Approximation and Projection (UMAP), pROC, and ggplot2 packages. The baseline characteristics were presented as mean ± SD for continuous variables and as number (%) for categorical variables. Unsupervised clustering based on 105 DEPs was performed by UMAP. The Wilcoxon rank-sum test was used to assess the statistical significance of differences between groups. Two-tailed *p* values <0.05 were considered indicative of statistical significance. ROC-AUCs were calculated by R package pROC. The Mann–Whitney U test or the Kruskal–Wallis rank-sum test was used for nonnormal data comparison. Fisher’s exact test was used to evaluate differences between the proportion of PAIM and lmCRC staining positive and negative for each marker. The differences between rates were tested by χ^2^ or Fisher’s exact tests, if appropriate. Logistic regression was used to model PAIM as a function of immunostaining. The corresponding ROC curves were plotted for different combinations of immunostains, and the areas under these correlated ROC curves were compared using the nonparametric approach of DeLong *et al.* (39)

## Results

### Clinicopathological Characteristics of the Study Participants

The study included a discovery cohort of 22 patients with PAIM and 17 patients with lmCRC, as well as a validation cohort of 11 PAIM and 19 lmCRC patients (Fig. 1). The clinicopathological characteristics of both cohorts were similar, with no significant differences in sex, age at diagnosis, smoking status, or tumor location between the PAIM and lmCRC patients (Table 1). In the validation cohort, tumors of PAIM were larger than those of lmCRC (*p* = 0.005 by Kruskal–Wallis rank-sum test). However, this finding did not apply to the discovery cohort, possibly due to the inclusion of biopsy samples in the validation cohort.

**Table 1:** Baseline characteristics of patients with PAIM and lmCRC

Features	Discovery cohort		Validation cohort		Total	
	PAIM	lmCRC	PAIM	lmCRC	PAIM	lmCRC
	N = 22	N = 17	N = 11	N = 19	N = 33	N = 36
Sex — n/N (%)						
Male	9/22 (40.9)	6/17 (35.3)	7/11 (63.6)	13/19 (68.4)	20/33 (60.6)	24/36 (66.7)
Female	13/22 (59.1)	11/17 (64.7)	4/11 (36.4)	6/19 (31.6)	13/33 (39.4)	12/36 (33.3)
Age at diagnosis — mean (SD) (yr)	62.91 (7.88)	58.47 (8.46)	66.18 (12.24)	60.05 (7.34)	64.00 (9.49)	59.31 (7.82)
Smoker — n/N (%)						
Yes	12/22 (54.5)	5/17 (29.4)	4/11 (36.4)	5/19 (26.3)	16/33 (48.5)	10/36 (27.8)
No	10/22 (45.5)	12/17 (70.6)	7/11 (63.6)	14/19 (73.7)	17/33 (51.5)	26/36 (72.2)
Tumor location — n/N (%)^a^						
Left superior lobe	5/22 (22.7)	1/17 (5.9)	1/10 (10.0)	2/19 (10.5)	6/32 (18.2)	3/36 (8.4)
Left inferior lobe	8/22 (36.4)	5/17 (29.4)	1/10 (10.0)	2/19 (10.5)	9/32 (27.3)	7/36 (19.4)
Right superior lobe	5/22 (22.7)	3/17 (17.6)	4/10 (40.0)	3/19 (15.8)	9/32 (27.3)	6/36 (16.7)
Right middle lobe	0/22 (0.0)	3/17 (17.6)	0/10 (0.0)	0/19 (0.0)	0/32 (0.0)	3/36 (8.3)
Right inferior lobe	4/22 (18.2)	3/17 (17.6)	3/10 (30.0)	4/19 (21.1)	7/32 (21.2)	7/36 (19.4)
Both lungs	0/22 (0.0)	2/17 (11.8)	1/10 (10.0)	8/19 (42.1)	1/32 (3.0)	10/36 (27.8)
Tumor size — median[IQR] (cm)	2.50 [1.77, 3.88]	2.50 [2.00, 3.50]	5.55 [4.23, 7.63]	2.60 [2.00, 3.30]	3.05 [2.00, 4.38]	2.50 [2.00, 3.45]
Tumor staging^b^						
I	13/22 (59.1)	0/17 (0.0)	0/11 (0.0)	0/19 (0.0)	13/33 (39.4)	0 (0.0)
II	3/22 (13.6)	0/17 (0.0)	1/11 (9.1)	0/19 (0.0)	4/33 (12.1)	0 (0.0)
III	3/22 (13.6)	0/17 (0.0)	2/11 (18.2)	0/19 (0.0)	5/33 (15.2)	0 (0.0)
IV	3/22 (13.6)	17/17 (100.0)	8/11 (72.7)	19/19 (100.0)	11/33 (33.3)	36 (100.0)
TTF-1 — n/N (%)						
Negative	13/22 (59.1)	16/17 (94.1)	7/11 (63.6)	16/19 (84.2)	20/33 (60.6)	32/36 (88.9)
Positive	9/22 (40.9)	1/17 (5.9)	4/11 (36.4)	3/19 (15.8)	13/33 (39.4)	4/36 (11.1)
NapsinA — n/N (%)^a^						
Negative	13/21 (61.9)	17/17 (100.0)	7/10 (70.0)	19/19 (100.0)	20/31 (64.5)	36/36 (100.0)
Positive	8/21 (38.1)	0/17 (0.0)	3/10 (30.0)	0/19 (0.0)	11/31 (35.5)	0/36 (0.0)
CK7 — n/N (%)						
Negative	1/22 (4.5)	15/17 (88.2)	0/11 (0.0)	14/19 (73.7)	1/33 (3.0)	29/36 (80.6)
Positive	21/22 (95.4)	2/17 (11.8)	11/11 (100.0)	5/19 (26.3)	32/33 (97.0)	7/36 (19.4)
CK20 — n/N (%)						
Negative	12/22 (54.5)	0/17 (0.0)	8/11 (72.7)	2/19 (10.5)	20/33 (60.6)	2/36 (5.6)
Positive	10/22 (45.5)	17/17 (100.0)	3/11 (27.3)	17/19 (89.5)	13/33 (39.4)	34/36 (94.4)
Villin — n/N (%)						
Negative	0/22 (0.0)	0/17 (0.0)	1/11 (9.1)	0/19 (0.0)	1/33 (3.0)	0/36 (0.0)
Positive	22/22 (100.0)	17/17 (100.0)	10/11 (90.9)	19/19 (100.0)	32/33 (97.0)	36/36 (100.0)
CDX2 — n/N (%)						
Negative	11/22 (50.0)	0/17 (0.0)	2/11 (18.2)	1/19 (5.3)	13/33 (39.4)	1/36 (2.8)
Positive	11/22 (50.0)	17/17 (100.0)	9/11 (81.8)	18/19 (94.7)	20/33 (60.6)	35/36 (97.2)

Abbreviations: IQR, interquartile range; lmCRC, lung metastatic colorectal cancer; PAIM, primary lung adenocarcinomas with intestinal or mucinous differentiation.

a Some patients were not tested.

b American Joint Committee on Cancer (AJCC) eighth edition.

Napsin A, TTF-1, and CK7 are all markers for lung adenocarcinoma in clinical practice. Napsin A was positively expressed in only 35.5% (11/31) of PAIM patients and negatively in all of lmCRC patients (36/36). TTF-1 was expressed in 39.4% (13/33) of PAIM patients, but also in 11.1% (4/36) of lmCRC patients. Similarly, CK7 staining was positive in 97.0% (32/33) of PAIM patients, while 19.4% (7/36) of lmCRC patients also displayed positive staining for this marker. In terms of intestinal markers, nearly all lmCRC patients displayed positive staining of CK20 (34/36, 94.4%), Villin (36/36, 100.0%), and CDX2 (35/36, 97.2%). In contrast, the expression of these markers varied among PAIM patients, posing a challenge to diagnosis. Specifically, positive staining of CK20 (13/33, 39.4%), Villin (32/33, 97.0%), and CDX2 (20/33, 60.6%) was observed among PAIM patients (Table 1).

### Proteomics Analysis of PAIM and lmCRC in the Discovery Cohort

**Supplemental Figure S1:**
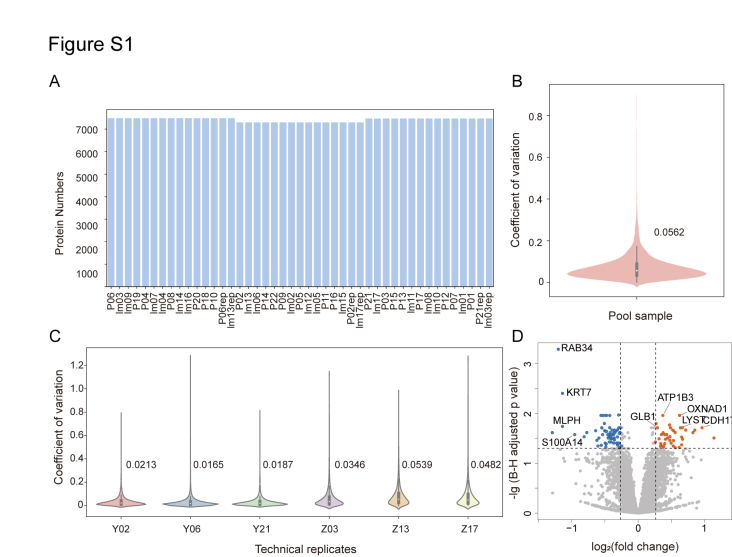
**The quality control and differential analysis of the proteomic data.***A*, the number of the identified proteins in each sample of the discovery cohort. The reproducibility of the proteomic data among the common pool samples (*B*) and between technical replicates (*C*). *D*, the *volcano plot* represents the differential analysis of proteomic data between the PAIM and lmCRC groups. lmCRC, lung metastatic colorectal cancer; PAIM, primary lung adenocarcinomas with intestinal or mucinous differentiation.

To identify potential biomarkers to distinguish between PAIM and lmCRC, we performed TMT-labeled quantitative proteomics using 39 FFPE tumors samples (22 PAIM and 17 lmCRC patients of the discovery cohort) obtained through lung resection surgery. A total of 7871 proteins were quantified, while the numbers of the quantified proteins in the three batches were 7490, 7301, and 7472, respectively, (supplemental Fig. S1*A* and supplemental Table S4). The CVs between each pair of technical replicates and among all common pools were all below than 20%, indicating high degree of reproducibility of protein quantification (supplemental Fig. S1, B and C).

**Fig. 2:**
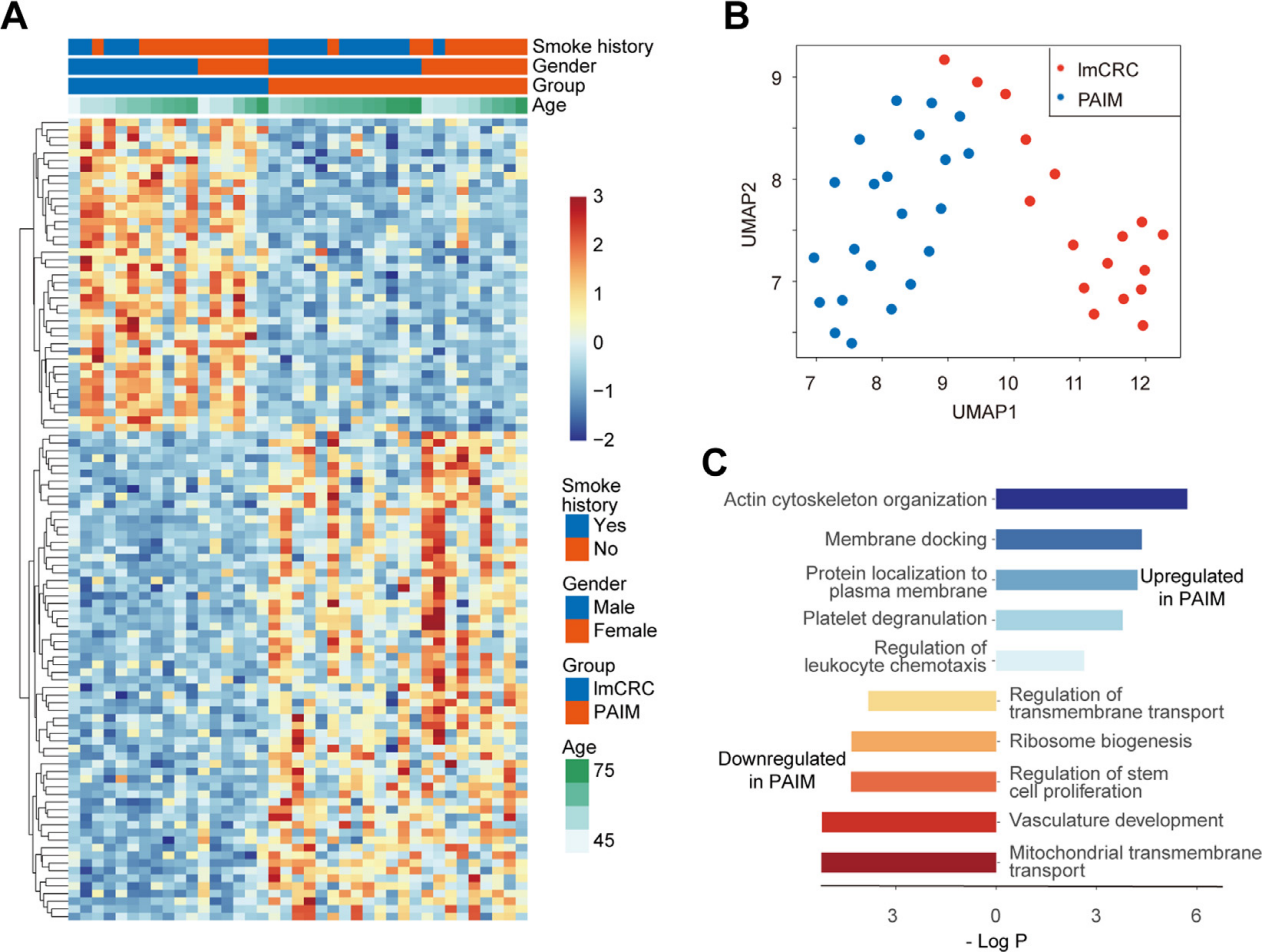
**Differentially expressed proteins between the PAIM and lmCRC.***A*, the heatmap represents the expression of 105 DEPs between the lmCRC and PAIM. The *color bar* indicates *Z*-score normalized protein ratio from TMT experiments. *B*, unsupervised clustering based on 105 DEPs by Uniform Manifold Approximation and Projection (UMAP). *C*, the enriched pathways for significantly upregulated and downregulated proteins in PAIM by Metascape. lmCRC, lung metastatic colorectal cancer; PAIM, primary lung adenocarcinomas with intestinal or mucinous differentiation; TMT, tandem mass tagging.

We identified 105 DEPs between PAIM and lmCRC groups (B–H adjusted *p* value <0.05 and |log_2_FC| > 0.25), with 41 proteins downregulated and 64 upregulated in PAIM compared to lmCRC (supplemental Figs. S2A and S1D, and supplemental Tables S5 and S6). UMAP clustering based on these 105 DEPs well separated the PAIM and lmCRC groups, whereas several samples between the groups are very close, reflecting similarities between the two groups (Fig. 2*B*). We then analyzed enriched pathways for significantly upregulated and downregulated proteins in PAIM group using Metascape. The upregulated proteins of PAIM group are involved in actin cytoskeleton organization, platelet degranulation, and regulation of leukocyte chemotaxis, while downregulated ones are involved in mitochondrial transmembrane transport, vasculature development, and stem cell proliferation (Fig. 2*C*).

### Selection of Diagnostic Protein Biomarkers in the Discovery Cohort

**Fig. 3:**
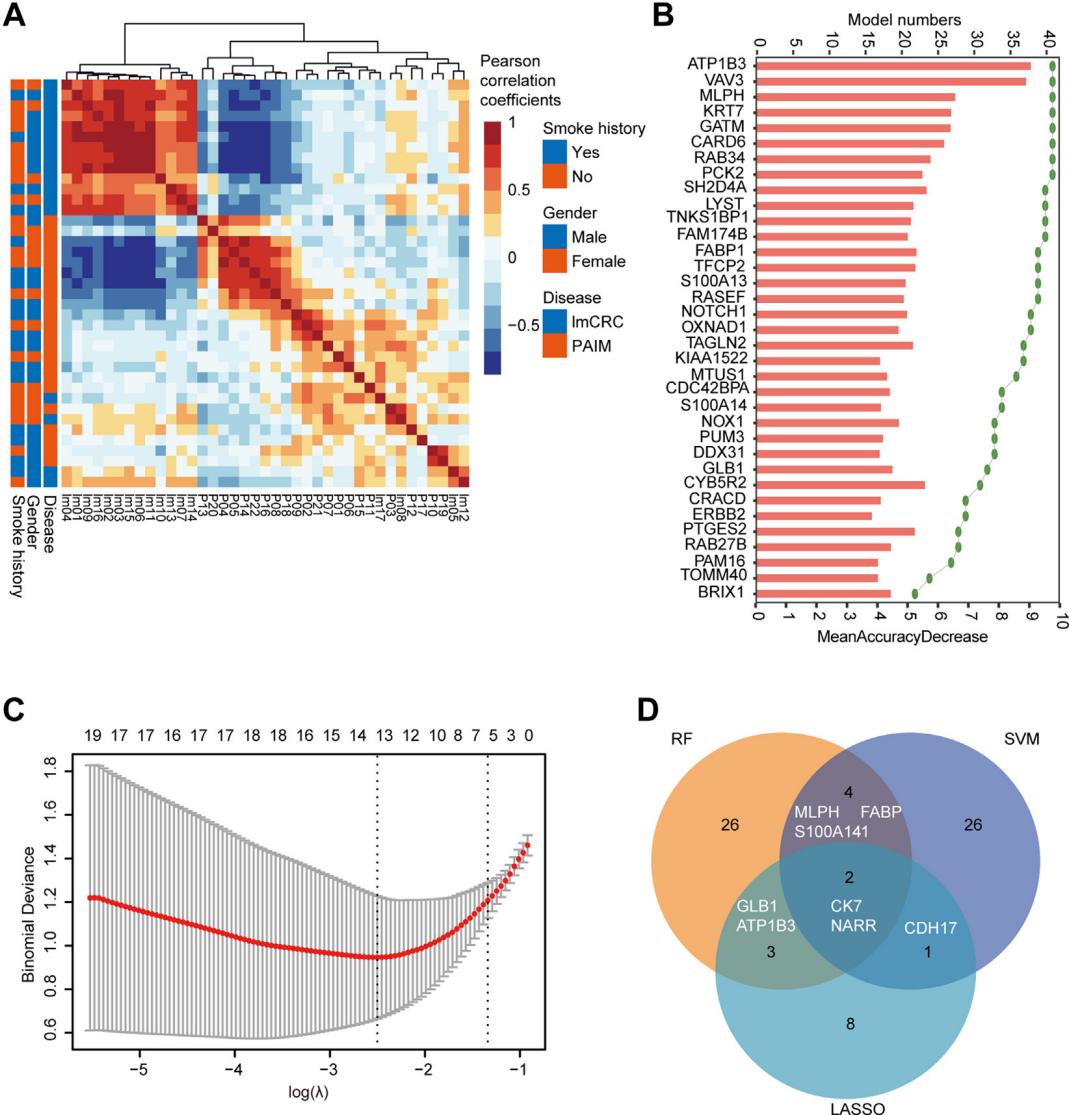
**Protein selection to distinguish the PAIM and lmCRC.***A*, unsupervised hierarchical clustering heatmap of Pearson correlation coefficients between every two samples based on the 33-protein SVM classifier. *B*, the protein features selected by random forest. The proteins were ranked by the inclusion frequency among 41 selected models. The *green dots* represent the inclusion frequency among 41 models, while the *bars* represent the average of mean decrease accuracy among them. *C*, the LASSO method showed a cross validation curve with binominal deviance along the lambda for shrinkage of coefficients. *D*, proteins with high ranking in all algorithms are displayed in *overlapping circles*. Proteins in *red* indicate highly expressed proteins in PAIM patients, while those in *green* indicate highly expressed proteins in lmCRC patients. LASSO, Least Absolute Shrinkage and Selection Operator; lmCRC, lung metastatic colorectal cancer; PAIM, primary lung adenocarcinomas with intestinal or mucinous differentiation; SVM, support vector machine.

To determine the most important proteins for distinguishing between PAIM and lmCRC, we employed three different algorithms, namely SVM, random forest, and LASSO. By SVM and cross-validation, we selected a minimum classifier, which provided the lowest error after six iterations of sampling. The final SVM classifier consisted of 33 proteins with the highest inclusion frequency, which well segregated the PAIM and lmCRC groups, except for a mixed clustering of five PAIM and three lmCRC samples (Fig. 3*A*). These samples in mixed clustering were located close in the UMAP by aforementioned DEPs, emphasizing the challenges to differentiate these two groups. For Random Forest, we selected 41 models with both AUC and accuracy of 1 and identified 35 proteins with high inclusion frequency in these models (Fig. 3*B*). By LASSO, one standard error of minimum lambda from a 10-fold cross-validation and minimum lambda were used to provide the stringent selection of biomarkers (Fig. 3*C*). This yielded a final set of 14 protein biomarkers.

We then combined the results of the three algorithms to obtain a set of ten candidate proteins that were consistently identified as important in distinguishing PAIM and lmCRC by at least two algorithms. Specifically, CDH17, ATP1B3, GLB1, OXNAD1, LYST, and FABP1 were found to be highly expressed in lmCRC, while NARR, MLPH, S100A14, and CK7 were highly expressed in PAIM (Fig. 3*D* and supplemental Table S7). These proteins may serve as potential biomarkers for differentiating between PAIM and lmCRC.

### IHC Validation of Protein Biomarkers in the Discovery Cohort

**Fig. 4:**
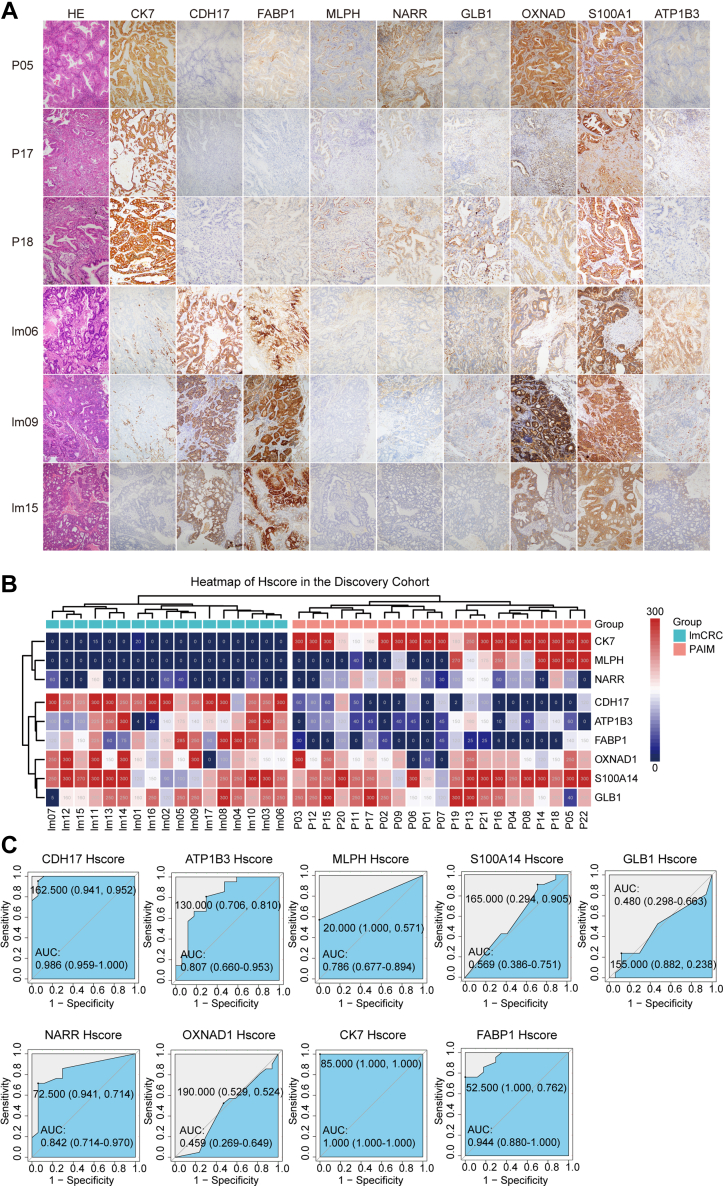
**IHC validation of protein biomarkers in the discovery cohort.***A*, images taken from PAIM and lmCRC tumor sections showing absent and present staining for CDH17, ATP1B3, GLB1, OXNAD1, FABP1, NARR, MLPH, S100A14, and CK7. *B*, immunostaining of candidate biomarkers, including CDH17, ATP1B3, GLB1, OXNAD1, FABP1, NARR, MLPH, S100A14, and CK7, is presented based on Hscore. The *red color* indicates a higher expression level, and the *blue* indicates a lower expression level. *C*, histogram comparing Hscore between two groups and ROC curves for protein biomarkers in the discovery cohort. AUC, area under curve; Hscore, histological score; IHC, immunohistochemical; lmCRC, lung metastatic colorectal cancer; ROC, receiver operating characteristic curve.

To validate the findings from proteomics analysis, IHC was performed on nine candidate proteins because of the lack of commercially available antibodies for LYST. The IHC analysis was conducted in the discovery cohort, and Hscore was used to measure protein expression levels. The results showed that CDH17 (AUC [95% CI], 0.986 [0.959–1.000], *p* < 0.01), FABP1 (AUC [95% CI], 0.944 [0.880–1.000], *p* < 0.01), and ATP1B3 (AUC [95% CI], 0.807 [0.660–0.953], *p* < 0.01) were significantly highly expressed in lmCRC patients, while NARR (AUC [95% CI], 0.842 [0.714–0.970], *p* < 0.01), MLPH (AUC [95% CI], 0.786 [0.677–0.894], *p* < 0.01), and CK7 (AUC [95% CI], 1.000 [1.000–1.000], *p* < 0.01) were significantly highly expressed in PAIM patients (Fig. 4 and supplemental Table S8). Moreover, hierarchal cluster analysis showed that patients in the discovery cohort were well separated into two clusters based on the Hscore of the biomarkers (Fig. 4*B*). Based on the comprehensive consideration of sensitivity and specificity, five protein biomarkers including CDH17, CK7, MLPH, FABP1, and NARR were selected for further verification. These results suggest that these five protein biomarkers may have potential clinical relevance in distinguishing between PAIM and lmCRC.

### IHC Validation of Protein Biomarkers in an Independent Cohort

**Fig. 5:**
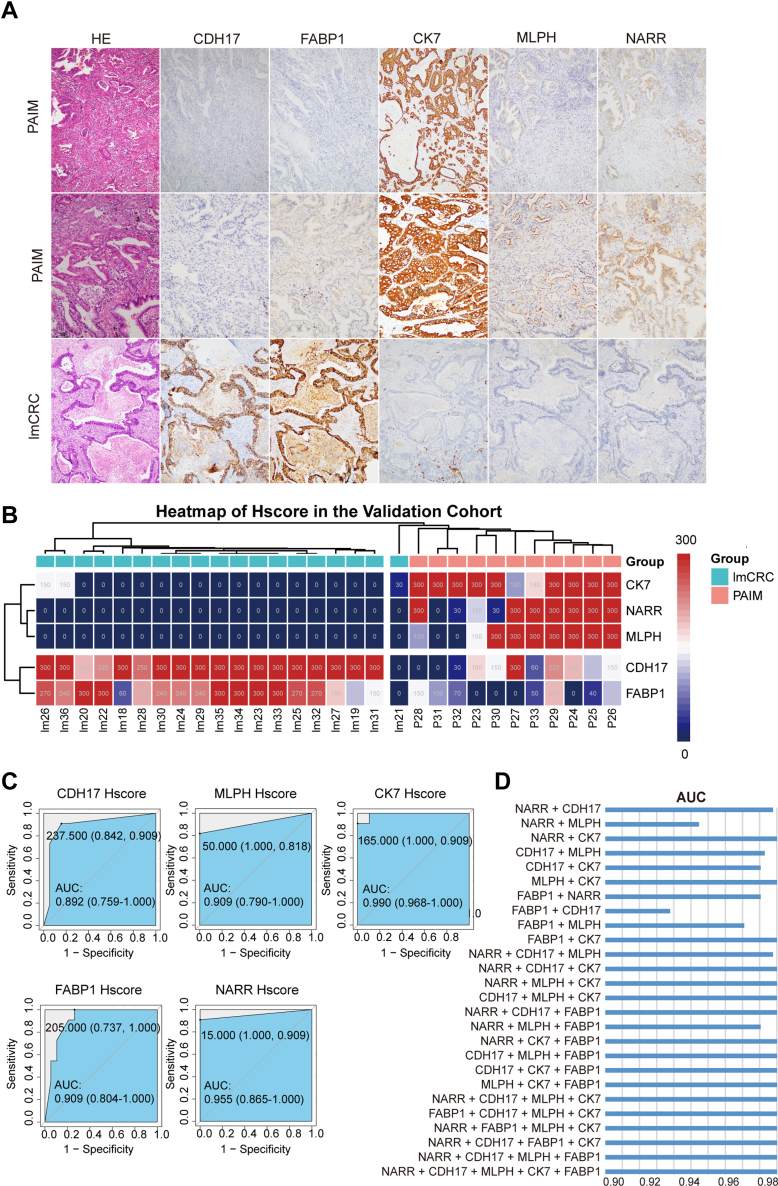
**IHC validation of protein biomarkers in the validation cohort.***A*, images taken from PAIM and lmCRC tumor sections showing absent and present staining for CK7, MLPH, CDH17, NARR, and FABP1. The *first row* represents primary lung adenocarcinomas with intestinal differentiation, while the *second row* represents primary lung adenocarcinomas with intestinal and mucinous differentiation. The images in the *first* and *third row* are 100× magnification, while the second row is 200× magnification. *B*, immunostaining of candidate biomarkers, including CDH17, FABP1, NARR, MLPH, and CK7, is presented based on Hscore. *C*, histogram comparing Hscore between two groups and ROC curves for protein biomarkers in the validation cohort. The *red color* indicates a higher expression level, and, the *blue* indicates a lower expression level. *D*, the diagnostic performance of the combination of biomarkers. AUC, area under curve; Hscore, histological score; IHC, immunohistochemical; lmCRC, lung metastatic colorectal cancer; PAIM, primary lung adenocarcinomas with intestinal or mucinous differentiation; ROC, receiver operating characteristic curve.

To validate the performance of protein biomarkers identified in the discovery cohort, an independent cohort of 11 PAIM and 19 lmCRC patients was collected for validation. By Hscore, CDH17 (AUC [95% CI], 0.892 [0.759–1.000], *p* < 0.01), and FABP1 (AUC [95% CI], 0.909 [0.804–1.000], *p* < 0.01) were significantly highly expressed in lmCRC patients, while NARR (AUC [95% CI], 0.955 [0.865–1.000], *p* < 0.01), MLPH (AUC [95% CI], 0.909 [0.790–1.000], *p* < 0.01), and CK7 (AUC [95% CI], 0.990 [0.968–1.000], *p* < 0.01) were significantly highly expressed in PAIM patients (Fig. 5 and supplemental Table S9).

Moreover, hierarchal cluster analysis also showed that patients in the validation cohort were well divided into two clusters based on the Hscore of the biomarkers, while one lmCRC patient was misclustered into PAIM (Fig. 5*B*). To further evaluate the potential clinical usefulness of these markers, multimarker combinations were evaluated using nominal logistic regression. Given that individual markers worked well, the combination of biomarkers naturally performed very well with AUC values >0.93 (Fig. 5*D* and supplemental Table S10).

Additionally, we conducted a comparative analysis of the diagnostic performance of both newly discovered markers and their combinations, as well as traditional markers and their combinations, in both the discovery and validation cohorts. The results indicated that CK7 exhibited the best diagnostic performance, followed by the four newly discovered markers (NARR, CDH17, MLPH, and FABP1), which outperformed other conventional markers (CDX2, Villin, CK20, NapsinA, and TTF-1) (supplemental Table S11). Furthermore, the combination of newly discovered markers demonstrated diagnostic performance comparable to that of the combination containing CK7 (supplemental Table S11).

Taken together, these findings suggest that the identified protein biomarkers, including CDH17, FABP1, NARR, and MLPH, may have potential clinical utility in accurately distinguishing between PAIM and lmCRC.

## Discussion

Due to their overlapping histomorphological, IHC, and genetic characteristics, it is difficult to distinguish between PAIM and lmCRC. Current differential diagnosis between PAIM and lmCRC mainly depends on careful clinical history, imaging, and pathological examinations, which is time and labor consuming. Even so, the differential diagnosis remains ambiguous in some cases, thus there is an urgent need to discover reliable biomarkers. In this study, we used LC-MS/MS to characterize proteomes of PAIM and lmCRC. Then three machine learning algorithms (SVM, random forest, and LASSO) were utilized to screen for differential proteins with diagnostic significance. The identified candidate proteins (CDH17, FABP1 highly expressed in lmCRC; NARR, MLPH, CK7 highly expressed in PAIM) were robustly validated in an independent cohort by IHC.

CK7, a member of the keratin family, is commonly used as a marker for primary lung adenocarcinoma, as well as to differentiate between primary and metastatic carcinoma (40, 41). Our study also identified CK7 as a potential biomarker for distinguishing PAIM from lmCRC, with all machine learning algorithms picking it out and IHC validating its expression, which also reflected the reliability of our research. Moreover, CK7 showed the best diagnostic performance in our research.

CDH17 is a cadherin protein that plays a role in the gastrointestinal tract and pancreatic ducts and has been linked to cancer metastasis (42, 43). Recent research has shown that CDH17 was a specific and more sensitive marker than those typically used ones (CK20 and CDX2) for gastrointestinal tract tumors (44). Consistently, in our results, CDH17 was highly expressed in lmCRC patients. Furthermore, CDH17 chimeric antigen receptor T cells have also been found to effectively eradicate CDH17-expressing neuroendocrine tumors and gastric, pancreatic and CRCs in either tumor xenograft or autochthonous mouse models (45). Notably, CDH17 chimeric antigen receptor T cells did not attack normal intestinal epithelial cells to cause toxicity, likely because CDH17 was localized only at the tight junction between normal intestinal epithelial cells (45). Hence, CDH17 represented a class of previously unappreciated tumor-associated antigens in gastrointestinal tract tumors that was negatively expressed in healthy tissues.

FABP1 is a fatty acid–binding protein, which is critical for fatty acid uptake and intracellular transport. It plays an important role in regulating lipid metabolism and signaling pathways (46, 47). A recent study analyzed the protein expression of FABP1 among 150 different tumor types containing 17,071 samples using a tissue microarray and found that the highest FABP1 positivity rates were observed in colorectal adenomas (86%), in colorectal adenocarcinomas (71.1%), and in hepatocellular carcinomas (65.3%), followed by mucinous carcinoma of the ovary (34.6%), cholangiocarcinoma (21.6%), and various adenocarcinomas from the digestive tract (10–23%) (48). Meanwhile, FABP1 staining was not seen in 169 primary adenocarcinomas of the lung (48). These results suggest that positive FABP1 immunostaining in an adenocarcinoma in the lung may indicate an extrapulmonary origin and favor metastases derived from colorectal carcinomas or other cancers of the gastrointestinal tract. Similarly, FABP1 was identified as one of verified markers for differential diagnosis between PAIM and lmCRC in our data. Conversely, considering the low FABP1 positivity rate in colorectal adenocarcinomas (71%) and the even lower frequency in other gastrointestinal adenocarcinomas, a negative FABP1 staining of an adenocarcinoma in the lung cannot indicate a pulmonary origin (48), consistent with our findings that FABP1 can also be negative in lmCRC patients.

MLPH is an important component involved in the transport of melanin, which contributes to visible pigmentation of hair and skin (49, 50). While its role in cancer is not well understood, recent research has found that MLPH is associated with tumor development and metastasis in skin, breast, and prostate cancer (51, 52, 53, 54, 55). Meanwhile, several studies demonstrated that MLPH was highly expressed in lung adenocarcinoma but not in squamous cell carcinoma, indicating that MLPH could be a potential marker for differential diagnosis (56, 57, 58).

NARR, an isoform of RAB34, is small GTPases involved in protein transport and is associated with many cancers (59, 60, 61, 62, 63, 64). For example, recent studies have found that RAB34 was downregulated in CRC (63) but upregulated in the non-small cell lung cancer (64) when compared with nonmalignant tissues. RAB34 expression is also linked with poor prognosis in CRC (63). In line with these findings, our data found that the expression of NARR is significantly higher in the PAIM group than the lmCRC group and verified its diagnostic significance for these two groups.

In this study, we identified the diagnostic protein biomarkers to distinguish PAIM from lmCRC based on proteomics. To date, previous studies using genomics and transcriptomics have not been effective in identifying diagnostic biomarkers that can differentiate between the two types of cancer, since they shared similar genetic alterations (14, 15, 16, 17, 18). In addition, diagnostic classifiers based on DNA methylation profiles were effective in discriminating enteric-type adenocarcinoma from lmCRC (19, 20). However, the differentially methylated regions screened by two research teams hardly share the same sites (19, 20). This discrepancy may be attributed to the inherent limitations of epigenetic analysis itself, as epigenomic data is highly dependent on the platform used in the analysis. Moreover, IMA has been found to share similar epigenetic and RNA expression patterns with upper gastrointestinal cancers (20, 21). Thus, this comprehensive analysis of proteome of PAIM and lmCRC provides a new approach to understand the biology of carcinomas and identifying novel diagnostic biomarkers. On the other hand, accurate identification and sampling of typical tumor areas with intestinal or mucinous differentiation allowed us to minimize the impact of stromal, immune, and other cell types present in the microenvironment on the analysis of low abundance proteins. It could potentially enhance diagnostic accuracy and enable the discovery of novel biomarkers. In addition, for high-dimensional proteomic data, three machine learning algorithms were utilized to reduce dimensionality and extract key molecular features with diagnostic significance. Although proteomics technologies and protocols become more and more accessible and may have potential for direct integration into clinical workflows (65), the current first-line approach for classification is still IHC-based. Therefore, we further validated protein biomarkers by IHC staining and robust biomarkers were subsequently picked out. Additionally, the validation cohort was composed of both surgical and biopsy samples, aligning better with the requirements of clinical practice. It indicates that the diagnostic panel developed here has the potential to be applied to biopsy samples.

Interestingly, one special case in our study caught our attention since the diagnosis process was full of plot reversal. This patient was initially classified as PAIM when enrolled in the validation cohort since only pulmonary nodule was detected by PET/CT and the endoscopy with biopsy revealed a hyperplastic polyp. However, during the validation process, based on the IHC results of our developed protein biomarkers (NARR, negative; MLPH, negative; CK7, focal slightly positive; CDH17, diffuse moderately positive; FABP1, diffuse moderately positive), this patient was classified into the lmCRC group. Subsequently, this patient underwent another endoscopy with biopsy in March 2023, which confirmed the presence of adenocarcinoma. Surgical resection was later performed, and the postoperative pathological examination identified a poorly differentiated adenocarcinoma of the sigmoid colon. As a result, the patient was ultimately diagnosed with lmCRC, underscoring the clinical significance of our findings.

In this study, our dataset primarily focuses on lung adenocarcinomas with intestinal/enteric differentiation, supplemented by a small proportion of cases presenting mucinous or colloid morphology. Although these mucinous or colloid adenocarcinomas represent a limited number of cases, their significance should not be overlooked. These variants may present diagnostic challenges in their histopathology and IHC results, potentially leading to confusion with metastatic colorectal mucinous adenocarcinomas to the lungs. Despite the small sample size involved, our study provided insights based on proteomic analysis and IHC validation, which offer potential for discriminating these subtypes. Large sample data is still needed to further validate the above results.

There are several potential limitations of this research. First, due to the low incidence of PAIM, the sample size of the discovery cohort was relatively small as mentioned above. Therefore, we carefully selected biomarkers by integrating multiple algorithms and further validated them in an independent cohort. Second, FYST, a promising biomarker, failed to be assessed by IHC due to the lack of the commercially available antibodies. We could design antibody-targeting FYST and reassessed the diagnostic performance in the future. Third, the validation cohort also had a relatively small sample size, highlighting the need for further external validation through multicenter studies. Fourth, as lung adenocarcinoma biomarkers, the diagnostic performance of NARR and MLPH was not as robust as the classical marker CK7. However, the discovery of these two new biomarkers can assist in the differential diagnosis and improve the accuracy of the diagnosis. Overall, while there are limitations, this study contributes important findings toward the development of diagnostic biomarkers in the field of cancer research.

To the best of our knowledge, it is the first study to explore the proteomic features of PAIM and lmCRC and to investigate the differential protein biomarkers for their diagnosis. The protein biomarkers identified, such as CK7, CDH17, MLPH, FABP1, and NARR, can be used for differential diagnosis between the two cancers in primary healthcare institutions using IHC staining. On the basis of good diagnostic performance when using a single biomarker above, the combination of multiple markers has better results. Moreover, these proteins may serve as potential therapeutic targets in patients with PAIM. Further functional studies and multicenter validation studies are required to understand the mechanism of action and to generalize the findings to a larger patient population. Overall, this study has significant implications for improving the diagnosis and treatment of PAIM and lmCRC.

## Data Availability

All data/information are available in the manuscript and in the Supplementary material. The proteomics data are deposited in the PRIDE (PXD050416). Username: reviewer_pxd050416@ebi.ac.uk. Password: nXljfugo.

## Supplemental Data

This article contains supplemental data.

## Conflict of interest

The authors declare that they have no competing interests.
