# Supplementary material for: Proteomics-Derived Biomarker Panel Facilitates Distinguishing Primary Lung Adenocarcinomas With Intestinal or Mucinous Differentiation From Lung Metastatic Colorectal Cancer
Source: Mol Cell Proteomics. 2024 Apr 10;23(5):100766. doi: 10.1016/j.mcpro.2024.100766 (PMC11092395; doi:10.1016/j.mcpro.2024.100766)
Supplement: Supplemental Data [file mmc12.docx]

Supplementary Materials

Table of Contents

**Supplementary Figure S1:** The quality control and differential analysis of the proteomic data.

**Supplementary Table S1:** Details of clinical and pathological characteristics of patients with PAIM and lmCRC.

**Supplementary Table S2:** Detection batches and grouping information of proteomic analysis.

**Supplementary Table S3:** Antibodies used for immunohistochemistry.

**Supplementary Table S4:** Proteins quantified and detected in PAIM and lmCRC samples of the discovery cohort.

**Supplementary Table S5:** The expression of 41 proteins highly expressed in lmCRC.

**Supplementary Table S6:** The expression of 64 proteins highly expressed in PAIM.

**Supplementary Table S7:** The candidate proteins screened by three machine learning algorithms.

**Supplementary Table S8:** The diagnostic performance of candidate protein biomarkers in the discovery cohort.

**Supplementary Table S9:** The diagnostic performance of candidate protein biomarkers in the validation cohort.

**Supplementary Table S10:** The diagnostic performance of the combination of biomarkers in the validation cohort.

**Supplementary Table S11:** The comparison of diagnostic performance for newly discovered biomarkers and their combinations versus conventional biomarkers and their combinations in discovery and validation cohorts.
